# Kir6.2 channel activity is regulated by interaction of transmembrane domains 1 and 2 through I167 in the bundle‐crossing gate

**DOI:** 10.14814/phy2.70481

**Published:** 2025-08-01

**Authors:** Ryoko Kawashima, Charlotte Meller, Manabu Suzuki, Shigeki Kato, Shoichiro Horita, Shizu Hidema, Shingen Misaka, Mitsuaki Hosoya, Hayato Go, Kazuto Kobayashi, Heidi de Wet, Yuko Maejima, Kenju Shimomura

**Affiliations:** ^1^ Department of Bioregulation and Pharmacological Medicine Fukushima Medical University School of Medicine Fukushima Japan; ^2^ Department of Pediatrics Fukushima Medical University School of Medicine Fukushima Japan; ^3^ Dept of Physiology, Anatomy and Genetics University of Oxford Oxford UK; ^4^ Electron Bio‐Imaging Centre (eBIC), Diamond Light Source Harwell Science & Innovation Campus Didcot, Oxfordshire UK; ^5^ Research Complex at Harwell Harwell Science & Innovation Campus Didcot, Oxfordshire UK; ^6^ Department of Molecular Genetics Institute of Biomedical Sciences, Fukushima Medical University School of Medicine Fukushima Japan; ^7^ Department of Diabetes, Endocrinology and Metabolism Fukushima Medical University School of Medicine Fukushima Japan; ^8^ Department of Perinatology and Pediatrics for Regional Medical Support Fukushima Medical University School of Medicine Fukushima Japan

**Keywords:** K_ATP_ channel, Kir6.2, SUR

## Abstract

ATP‐sensitive potassium (K_ATP_) channel in pancreatic β‐cells is composed of four pore‐forming inward rectifier potassium (Kir) 6.2 subunits and four regulatory sulfonylurea receptor (SUR) 1 subunits and regulate insulin secretion. Kir6.2 consists of a N‐terminal region, an outer transmembrane helix (TM1), an intramembrane region that functions as a potassium selectivity filter, an inner transmembrane helix (TM2) that forms a bundle‐crossing gate, and a C‐terminal cytoplasmic domain. Mutations in the Kir6.2 subunit can cause neonatal diabetes with severe neurological features (DEND syndrome). The DEND syndrome‐inducing I167L mutation of Kir6.2 increases the open probability (*P*_o_) of the K_ATP_ channel. To investigate the gating mechanism impacted by this mutation in Kir6.2 alone, we used C‐terminus‐truncated Kir6.2 channels to ascertain the impact of I167 mutations on *P*_o_ in Kir6.2 channels in the absence of SUR1. We found that I167L and I167F mutations showed an increased *P*_o_ while the *P*_o_ of other mutations (I167A, I167V) were unchanged when compared to wild‐type channels. By mutating residues in TM1 (W68, L72, F75) that may interact with I167, we found that a double mutation of I167L and F75A normalized the *P*_o_. These results would suggest that I167 may play an important role in stabilizing the open state of Kir6.2 channels.

## 1 INTRODUCTION

**FIGURE 1:**
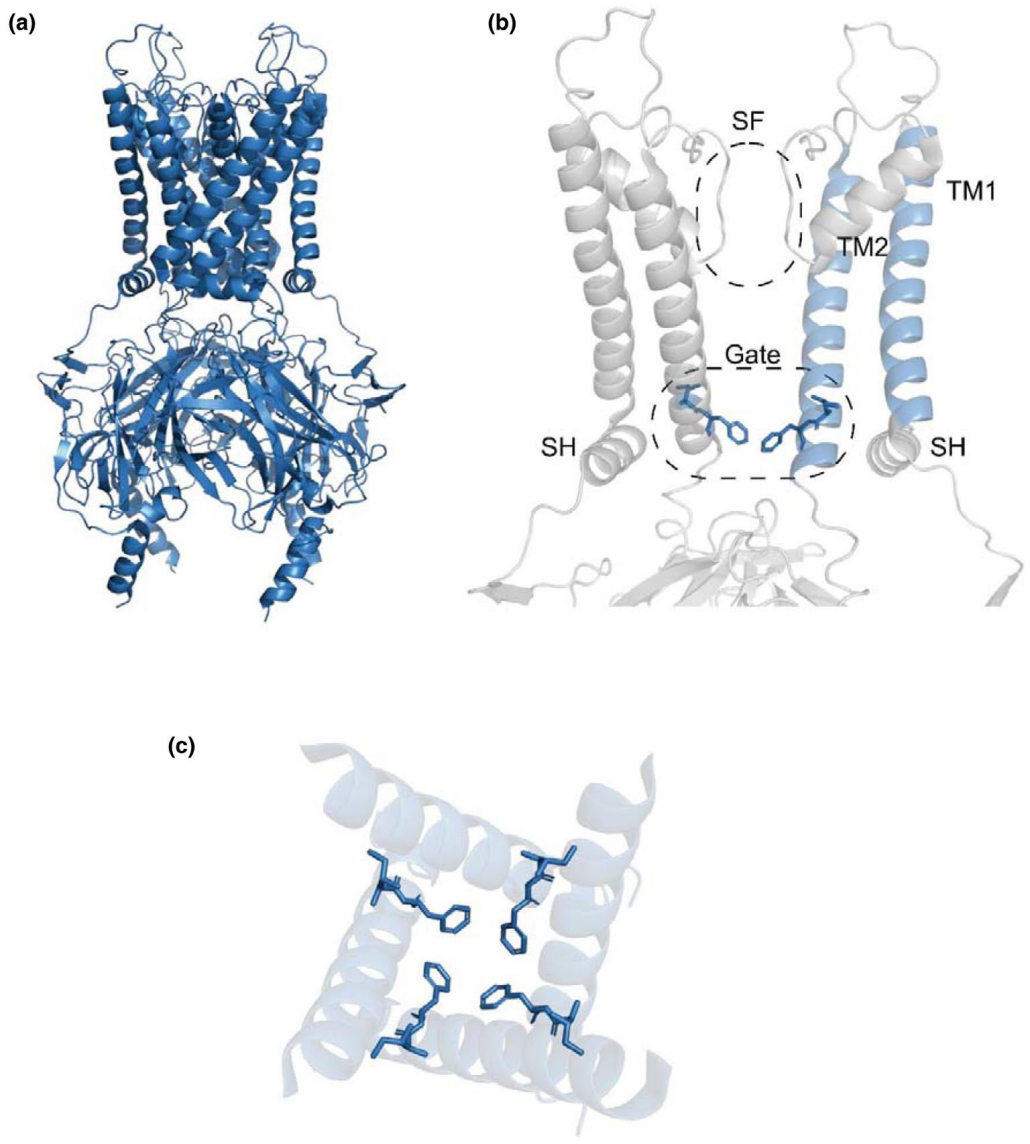
Structure of closed Kir6.2 (PDB: 6C3O). (a) View of the Kir6.2 tetramer. (b) The Kir6.2 channel with the bundle‐crossing gate (Gate), selectivity filter (SF), slide helix (SH), transmembrane helix 1 (TM1) and transmembrane helix 2 (TM2) labeled. F168 and I167 are shown as sticks. (c) The relative position of F168 and I167 in the channel pore viewed from the top.

ATP‐sensitive potassium (K_ATP_) channels function as intracellular nucleotide sensors that link cellular metabolism to excitability in tissues such as pancreatic β‐cells, brain, and muscle (Gribble & Ashcroft, 2000). The K_ATP_ channel complex in pancreatic β‐cells is composed of four pore‐forming inwardly rectifier potassium (Kir) 6.2 subunits and four regulatory sulfonylurea receptor (SUR) 1 subunits and plays a critical role in insulin secretion. Kir6.2 consists of an N‐terminal region, an outer transmembrane helix (TM1), an intramembrane region that functions as a potassium selectivity filter, an inner transmembrane helix (TM2) that forms a bundle‐crossing gate, and a C‐terminal cytosolic domain (Driggers & Shyng, 2023) (Figure 1). Elevation of glucose metabolism in pancreatic β‐cells results in an increase in intracellular ATP levels. Binding of ATP to Kir6.2 shuts the channel while binding of MgATP/ADP to the nucleotide‐binding domains of SUR1 activates the channel. In the β‐cell, an increase in intracellular ATP induced by metabolism promotes K_ATP_ channel closure resulting in membrane depolarization, opening of voltage‐gated Ca^2+^ channels, Ca^2+^ influx, and ultimately leading to exocytosis of insulin‐containing granules (Tarasov et al., 2004). Therefore, mutations in the K_ATP_ channel can cause neonatal diabetes (gain‐of‐function mutations) or hyperinsulinemia (loss‐of‐function mutations) (ElSheikh & Shyng, 2023; Gloyn et al., 2004; Shimomura & Maejima, 2017).

Recent studies revealed the high‐resolution cryoEM structure of the K_ATP_ channel complex, providing structural context to numerous functional studies of mutated K_ATP_ channels performed prior to the availability of a high‐resolution structure (Li et al., 2017; Martin, Yoshioka, et al. 2017). A large number of high‐resolution structures of mammalian Kir6.2/SUR K_ATP_ channels have now been reported in both the open and closed states, from different species and in the presence of ligands including ATP, ADP, and glibenclamide, as summarized in Table 1.

**TABLE 1:** Current published mammalian Kir6.2/SUR structures.

PDB	Year	Resolution	Conformation	Mutations	ATP/ADP	Ligands	Organisms	References
5TWV	2017	6.3	Closed	‐	ATP (Kir6.2)	Glibenclamide	Cricetus cricetus/rattus norvegicus	Martin, Yoshioka, et al. (2017)
5WUA	2017	5.6	Closed	‐	‐	‐	Mesocricetus auratus/Mus musculus	Li et al. (2017)
6BAA	2017	3.63	Closed	‐	ATP (Kir6.2)	Glibenclamide	Cricetus cricetus/rattus norvegicus	Martin, Kandasamy, et al. (2017)
6C3P	2017	5.6	Closed	‐	ATP (Kir6.2) ATP (SUR) ADP (SUR)	‐	Homo sapiens	Lee et al. (2017)
6C3O	2017	3.9	Closed	‐	ATP (Kir6.2) ATP (SUR) ADP (SUR)	‐	Homo sapiens	Lee et al. (2017)
5YKE	2018	4.11	Closed	‐	‐	Glibenclamide	Mesocricetus auratus/Mus musculus	Wu et al. (2018)
5YKF	2018	4.33	Closed	‐	ATP gamma S (Kir6.2 + SUR)	Glibenclamide	Mesocricetus auratus/Mus musculus	Wu et al. (2018)
5YKG	2018	4.57	Closed	‐	ATP gamma S (Kir6.2 + SUR)	Glibenclamide	Mesocricetus auratus/Mus musculus	Wu et al. (2018)
5YW8	2018	4.4	Closed	‐	ATP gamma S (Kir6.2 + SUR)	‐	Mesocricetus auratus/Mus musculus	Wu et al. (2018)
5YW9	2018	5	Closed	‐	ATP gamma S (Kir6.2 + SUR)	‐	Mesocricetus auratus/Mus musculus	Wu et al. (2018)
5YWA	2018	6.1	Closed	‐	ATP gamma S (Kir6.2 + SUR)	‐	Mesocricetus auratus/Mus musculus	Wu et al. (2018)
5YWC	2018	4.3	Closed	‐	ADP (Kir6.2 + SUR)	‐	Mesocricetus auratus/Mus musculus	Wu et al. (2018)
5YWB	2018	5.2	Closed	‐	ADP (Kir6.2 + SUR)	‐	Mesocricetus auratus/Mus musculus	Wu et al. (2018)
6JB1	2019	3.3	Closed	‐	ATP gamma S (Kir6.2 + SUR)	Repaglinide	Mesocricetus auratus/Mus musculus	Ding et al. (2019)
6PZ9	2019	3.65	Closed	‐	ATP (SUR)	Repaglinide	Cricetus cricetus/rattus norvegicus	Martin et al. (2019)
6PZA	2019	3.74	Closed	‐	ATP (SUR)	Glibenclamide	Cricetus cricetus/rattus norvegicus	Martin et al. (2019)
7S5T	2021	3.1	Open	Kir C166S G334D	‐	‐	Homo sapiens	Zhao and MacKinnon (2021)
7S5X	2021	3.7	Open	Kir C166S G334D	ATP (SUR) ADP (SUR)	‐	Homo sapiens	Zhao and MacKinnon (2021)
7S5Y	2021	3.9	Open	Kir C166S G334D	ATP (SUR) ADP (SUR)	‐	Homo sapiens	Zhao and MacKinnon (2021)
7S5Z	2021	3.9	Open	Kir C166S G334D	ATP (SUR) ADP (SUR)	‐	Homo sapiens	Zhao and MacKinnon (2021)
7S60	2021	3.7	Open	Kir C166S G334D	ATP (SUR) ADP (SUR)	‐	Homo sapiens	Zhao and MacKinnon (2021)
7S61	2021	4	Open	Kir C166S G334D	ATP (SUR) ADP (SUR)	‐	Homo sapiens	Zhao and MacKinnon (2021)
7W4O	2022	2.96	Pre‐open	Kir H175K	ATP (SUR) ADP (SUR)	E2H	Mesocricetus auratus/Mus musculus	Wang et al. (2022)
7W4P	2022	3.19	Closed	Kir H175K	ATP (SUR) ADP (SUR) ADP (Kir6.2)	E2H	Mesocricetus auratus/Mus musculus	Wang et al. (2022)
7TYS	2022	3.41	Closed	‐	ATP (SUR) ATP (Kir6.2)	Repaglinide	Cricetus cricetus/rattus norvegicus	Sung et al. (2022)
7TYT	2022	3.6	Closed	‐	ATP (SUR) ATP (Kir6.2)	Repaglinide	Cricetus cricetus/rattus norvegicus	Sung et al. (2022)
7U1E	2022	4.52	Closed	‐	ATP (SUR) ATP (Kir6.2)	‐	Cricetus cricetus/rattus norvegicus	Sung et al. (2022)
7U1Q	2022	3.9	Closed	‐	ATP (SUR) ATP (Kir6.2)	Repaglinide	Cricetus cricetus/rattus norvegicus	Sung et al. (2022)
7U1S	2022	3.8	Closed	‐	ATP (SUR) ATP (Kir6.2)	Repaglinide	Cricetus cricetus/rattus norvegicus	Sung et al. (2022)
7 U24	2022	3.58	Closed	‐	ATP (SUR) ATP (Kir6.2)	Glibenclamide	Cricetus cricetus/rattus norvegicus	Sung et al. (2022)
7U2X	2022	4.1	Closed	‐	ATP (SUR)	Carbamazepine	Cricetus cricetus/rattus norvegicus	Sung et al. (2022)
7U6Y	2022	7.4	Closed	‐	ATP (SUR) ATP (Kir6.2)	Glibenclamide	Cricetus cricetus/rattus norvegicus	Sung et al. (2022)
7U7M	2022	5.2	Closed	‐	ATP (SUR)	Carbamazepine	Cricetus cricetus/rattus norvegicus	Sung et al. (2022)
7UAA	2022	5.7	Closed	‐	ATP (SUR) ATP (Kir6.2)	‐	Cricetus cricetus/rattus norvegicus	Sung et al. (2022)
7UQR	2022	4.55	Closed	‐	‐	‐	Cricetus cricetus/rattus norvegicus	Sung et al. (2022)
8TI1	2024	2.9	Open	Kir6.2 Q52R	‐	PIP2	Mesocricetus auratus/Rattus norvegicus	Driggers & Shyng (2023)
8TI2	2024	3.28	Open	Kir6.2 Q52R	‐	PIP2	Mesocricetus auratus/Rattus norvegicus	Driggers & Shyng (2023)
9DFX	2024	4.1	Closed	‐	ATP (SUR) ATP (Kir6.2)	Aekatperone	Mesocricetus auratus/Rattus norvegicus	Elsheikh et al. (2025)

In 2007, we reported the gain‐of‐function mutation of Kir6.2, isoleucine to leucine at residue 167 (I167L), which causes neonatal diabetes with severe neurological disorders known as DEND syndrome (developmental delay, epilepsy and neonatal diabetes) (Shimomura et al., 2007). Functional studies revealed that K_ATP_ channels with the I167L mutation in Kir6.2 are less sensitive to inhibition by ATP combined with a higher open probability (*P*_o_ = 0.82) compared to the wild type (WT) channels. In pancreatic β‐cells, I167L mutations cause impaired insulin secretion and are expected to reduce the firing rate and transmitter release in neurons.

From the latest high‐resolution structure obtained for the K_ATP_ channel complex, I167 lies at the cytoplasmic end of the TM2 of Kir6.2, and the neighboring residue F168 is proposed to act as an intracellular gate for the channel (Figure 1). Taking both the existing electrophysiological data and the position of I167 into account, I167 may therefore be a key residue that regulates the opening and closing of the K_ATP_ channel.

Here, we specifically focus on the Kir6.2 channel pore to investigate the role of I167 in the regulation of channel activity and open probability, with an additional emphasis on the impact of nearby residues W68, L72, and F75 in TM1 on channel gating. The *P*_o_ of the Kir6.2 channel is regulated by dynamic conformational changes of the central channel pore. Understanding the molecular interactions that affect the opening and closing of the central channel pore, known as “gating,” can provide important information about the underlying mechanisms that underpin ion channel activity. This is particularly relevant to mutations causing DEND syndrome, which are often refractory to treatment. Current available medication relies on the binding of drugs to the regulatory SUR subunit to lower channel open probability and is often ineffective in the treatment of DEND patients where mutations uncouple Kir6.2 channel gating from regulation by the SUR1 subunit. In the present study, by focusing on residue I167 in TM2 in the absence of SUR1, we provide important insights for understanding the mechanism and interaction of TM2 and TM1 in regulating the opening and closing of the channel, which could provide valuable information for the design of new DEND drugs that do not target SUR1.

## 2 MATERIALS AND METHODS

### 2.1 HEK293T cell culture

Human embryonic kidney cell lines possessing SV40 large T antigens (HEK293T) were kindly provided by Prof. Kazuto Kobayashi (Fukushima Medical University) and cultured according to conventional methods. Briefly, HEK293T cells were cultured at 37°C in a humidified atmosphere of 5% CO_2_ in growth medium consisting of high‐glucose Dulbecco's modified Eagle's medium (DMEM; Wako, Osaka, Japan), 10% (v/v) heat‐inactivated fetal bovine serum (FBS) (Equitech Bio, Kerrville, TX, USA), 50 U/mL penicillin (Wako), and 50 μg/mL streptomycin (Wako). When the cells reached 70%–80% confluence, the cells were detached and subcultured with a cell density of 1.0–5.0 × 10^4^ cells/mL (8 mL) into a 10 cm petri dish. The cells were also split into a 6‐well plate with a cell density of 3.0 × 10^4^ cells/mL (2 mL) for viral infection and electrophysiological experiments.

### 2.2 Viral vector preparation

The plasmid pAAV vector encoded the EF1α promoter, 2A self‐cleaving peptide, red fluorescent protein (RFP) and woodchuck hepatitis virus posttranscriptional regulatory element (WPRE) sequences. The C‐terminally truncated Kir6.2 (Kir6.2ΔC36) was generated by using the wild‐type Kir6.2 cDNA as templates and primers 5′‐GTC GTG AAA TTC GGA TCC TCT AGA GTC GAC ATG CTC TCA AGA AAG GGG ATT‐3′ and 5′‐TAG CAG ACT TCC TCT GCC CTC TCC ACT GCC AGA TCT GTC CTC ATC CAG TTG‐3′. The amplified fragments of Kir6.2ΔC36 were incorporated into the pAAV vectors using primers 5′‐GGC AGT GGA GAG GGC AGA GGA AGT CTG CTA‐3′ and 5′‐GTC GAC TCT AGA GGA TCC GAA TTT CAC GAC‐3′ for homogeneous recombination, which was performed by using the in‐fusion HD cloning kit (Takara Bio, Shiga, Japan). Next, the Kir6.2ΔC36/I167L, Kir6.2ΔC36/I167A, Kir6.2ΔC36/I167V mutants encoding plasmids were constructed using 5′‐CTG ATG ATC AAC GCC ATA ATG CTG GGG TGT CTG TTC ATG AAA ACC GCA CAG GCT CAT AGG AGA‐3′ (Forward) and 5′‐TCT CCT ATG AGC CTG TGC GGT TTT CAT GAA CAG ACA CCC CAG CAT TAT GGC GTT GAT CAT CAG‐3′ (Reverse) in Kir6.2ΔC36/I167L; 5′‐CTG ATG ATC AAC GCC ATA ATG CTG GGG TGT GCA TTC ATG AAA ACC GCA CAG GCT CAT AGG AGA‐3′ (Forward) and 5′‐TCT CCT ATG AGC CTG TGC GGT TTT CAT GAA TGC ACA CCC CAG CAT TAT GGC GTT GAT CAT CAG‐3′ (Reverse) in Kir6.2ΔC36/I167A; 5′‐CTG ATG ATC AAC GCC ATA ATG CTG GGG TGT GTA TTC ATG AAA ACC GCA CAG GCT CAT AGG AGA‐3′ (Forward) and 5′‐TCT CCT ATG AGC CTG TGC GGT TTT CAT GAA TAC ACA CCC CAG CAT TAT GGC GTT GAT CAT CAG‐3′(Reverse) in Kir6.2ΔC36/I167V. Plasmids encoding the Kir6.2ΔC36/I167F and Kir6.2ΔC36/I167Q mutants were constructed using the Gene Synthesis Service from GenScript Japan Inc. The constructs were cloned into pAAV‐EF1α‐mKir6.2(ΔC36)‐2A‐cgfTag‐RFP vectors (Quote No: J869UZPVG0). The additional mutations W68A, L72A, F75A were introduced using 5′‐GAC GTG TTT ACG ACT CTT GTG GAT CTC AAA GCA CCT CAT ACC CTG CTG ATC TTT ACG ATG TCT‐3′ (Forward) and 5′‐AGA CAT CGT AAA GAT CAG CAG GGT ATG AGG TGC TTT GAG ATC CAC AAG AGT CGT AAA CAC GTC‐3′ (Reverse) in Kir6.2ΔC36/I167L/W68A and Kir6.2ΔC36/W68A; 5′‐ACT CTT GTG GAT CTC AAA TGG CCT CAT ACC GCA CTG ATC TTT ACG ATG TCT TTC CTT TGC AGC‐3′ (Forward) and 5′‐GCT GCA AAG GAA AGA CAT CGT AAA GAT CAG TGC GGT ATG AGG CCA TTT GAG ATC CAC AAG AGT‐3′ (Reverse) in Kir6.2ΔC36/I167L/L72A and Kir6.2ΔC36/L72A; 5′‐GAT CTC AAA TGG CCT CAT ACC CTG CTG ATC GCT ACG ATG TCT TTC CTT TGC AGC TGG CTG CTG‐3′ (Forward) and 5′‐CAG CAG CCA GCT GCA AAG GAA AGA CAT CGT AGC GAT CAG CAG GGT ATG AGG CCA TTT GAG ATC‐3′ (Reverse) in Kir6.2ΔC36/I167L/F75A and Kir6.2ΔC36/F75A. The viral production was performed as described in the previous study (Horita et al., 2021). Around 50% of confluent HEK293T cells grew in high‐glucose DMEM growth medium supplemented with 10%(v/v) FBS, 50 U/mL penicillin, and 50 μg/mL streptomycin were triple transfected with a pHelper vector (11,635 bp, Takara Bio), a pRC2‐mi342 vector (8189 bp, Takara Bio), and the above‐described pAAV vector encoding the Kir6.2 (WT) or mutated Kir6.2 gene plasmids, using an HBSP solution (5 mM HEPES) (DOJINDO, Cat. No. 346‐01373), 140 mM NaCl (Wako, Cat. No. 191‐01665), 0.75 mM Na_2_HPO_4_ (Nacalai tesque, Cat. No. 31723‐35), 5 mM KCl (Wako, Cat. No. 163–03545), and 6 mM D(+)‐Glucose (Wako, Cat. No. 041‐00595) at pH 7.1. After transfection for 72 h, cells were harvested by centrifugation (1000 g, 3 min), resuspended in TBS Buffer (25 mM Tris (Wako, Cat. No. 201‐06273), 137 mM NaCl, 2.68 mM KCl) at pH 7.4, and disrupted by freeze‐thaw cycles (three times). After disruption, benzonase (Novagen Millipore, Cat. No. 71205‐3CN) was added and incubated with DNaseI (Takara Bio, Cat. No. 2270A) for 30 min at 37°C. After centrifugation (10,000 g, 15 min), the centrifugation tubes with CsCl_2_ (Wako, Cat. No. 033‐25035), dissolved in phosphate‐buffered saline (PBS) and the supernatant were centrifuged (55,000*g*) for 24 h at 16°C. This CsC_l2_‐based purification step was repeated twice with two CsCl_2_ concentrations (1.24 and 0.55 g/mL). After gravity flow purification, each fraction was confirmed by reverse transcription polymerase chain reaction (RT‐PCR) using the WPRE sequence and dialyzed three times to replace the Cs‐containing buffer with PBS buffer. After concentrating approximately to 100 μL, the virus was frozen at −80°C. The genomic titer was determined by RT‐PCR.

### 2.3 Electrophysiology

The patch‐clamp technique was used to study single‐channel activity in inside‐out membrane patches, and all electrophysiological measurements were performed at room temperature (22–25°C) using an EPC‐800 patch clamp amplifier (HEKA Electronics, Lambrecht/Pfalz, Germany) and pCLAMP 10 software (Molecular Devices, CA, USA). Standard patch clamp techniques with inside‐out membrane patches were used to record K_ATP_ channel currents. The pipette solution contained (in mM): 140 KCl, 2.0 CaCl_2_, 10.0 HEPES at pH 7.4 with KOH. The composition of the bathing solution was (in mM): 110 KCl, 11 HEPES, 2 MgCl_2_, 11 EGTA, 1 CaCl_2_ at pH 7.2 with KOH. For the K_ATP_ channel recording, the patch membrane potential was held at −60 mV. *P*_o_s were determined from current records of 1 min duration.

### 2.4 Structure analysis

PyMOL molecular graphics system was used to analyze and generate figures of the closed (PDB: 6C3O) and open (PDB: 7S5T) conformations of human Kir6.2.

### 2.5 Statistical analysis

The data are presented as the mean ± standard deviation of the mean (SD) unless otherwise indicated. Student's *t*‐test was applied for analyzing significant differences. All statistical analyses were performed using ORIGIN software, version 10.14.6. A *p* value of <0.05 was considered statistically significant.

## 3 RESULTS

### 3.1 I167 mutated channels

**FIGURE 2:**
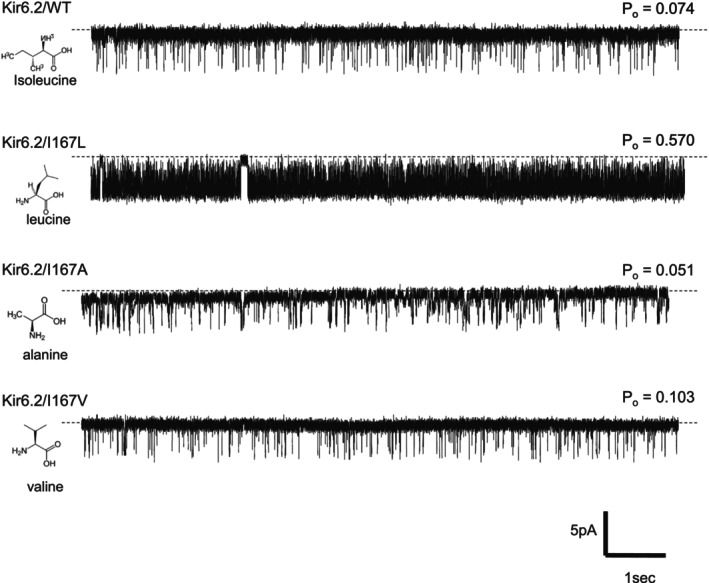
Single channel recordings of Kir6.2ΔC36 WT channels and I167L, I167A and I167V.

To investigate the effect of I167 on Kir6.2 activity in the absence of SUR1, all experiments were performed on 36 amino acid C‐terminally truncated Kir6.2 (Kir6.2ΔC36), which traffics to the membrane in the absence of SUR1, expressed in human embryonic kidney (HEK) cells using viral transfection (Tucker et al., 1997). Patch clamp recordings of channel activity show a significantly higher *P*_o_ for I167L compared to the WT, in the absence of ATP (*P*_o_: 0.074 ± 0.060 (*n* = 10) for Kir6.2ΔC36 vs. *P*_o_: 0.570 ± 0.239 (*n* = 13) for I167L; *p* < 0.00001) (Figure 2), which is consistent with previous studies using the whole K_ATP_ channel complex (Kir6.2+SUR1) expressed in *Xenopus oocytes* in the absence of ATP [*P*_o_: 0.27 ± 0.12 for WT vs. *P*_o_: 0.82 ± 0.11 for I167L] (Shimomura et al., 2007).

Next, we tested two alternative hydrophobic mutations of I167, I167A and I167V. The *P*_o_ values compared to WT (*P*_o_: 0.051 ± 0.036 (*n* = 7) for I167A and *P*_o_: 0.103 ± 0.113 (*n* = 6) for I167V) (Figure 2). Furthermore, we tested two mutants affecting hydrophobicity (I167Q) and side chain size (I167F) respectively. The *P*_o_ of I167Q was unchanged compared to WT (*P*_o_: 0.115 ± 0.054 [n = 6]) but the *P*_o_ was significantly increased for the I167F mutation compared to WT (*P*_o_: 0.406 ± 0.116 (n = 6); *p* < 0.00001 compared to WT) suggesting that side chain size, instead of charge, is important (Table 2).

**TABLE 2:** Channel Open potentials (P_o_) of WT Kir6.2 and mutants reported here.

Mutation	P_o_	*p*‐value
WT	0.074 ± 0.060	‐
I167L	0.570 ± 0.239	*p* < 0.00001 (vs. WT)
I167A	0.051 ± 0.036	‐
I167V	0.103 ± 0.113	‐
I167Q	0.115 ± 0.054	‐
I167F	0.406 ± 0.116	*p* < 0.00001 (vs. WT)
W68A	0.099 ± 0.045	‐
L72A	0.151 ± 0.079	*p* < 0.05 (vs. WT), *p* < 0.00001 (vs. I167L/L72A)
F75A	0.085 ± 0.074	‐
I167L/W68A	0.318 ± 0.199	*p* < 0.05
I167L/L72A	0.59 ± 0.246	*p* < 0.05
I167L/F75A	0.11 ± 0.12	*p* < 0.05

### 3.2 I167L and TM1 mutated channels

**FIGURE 3:**
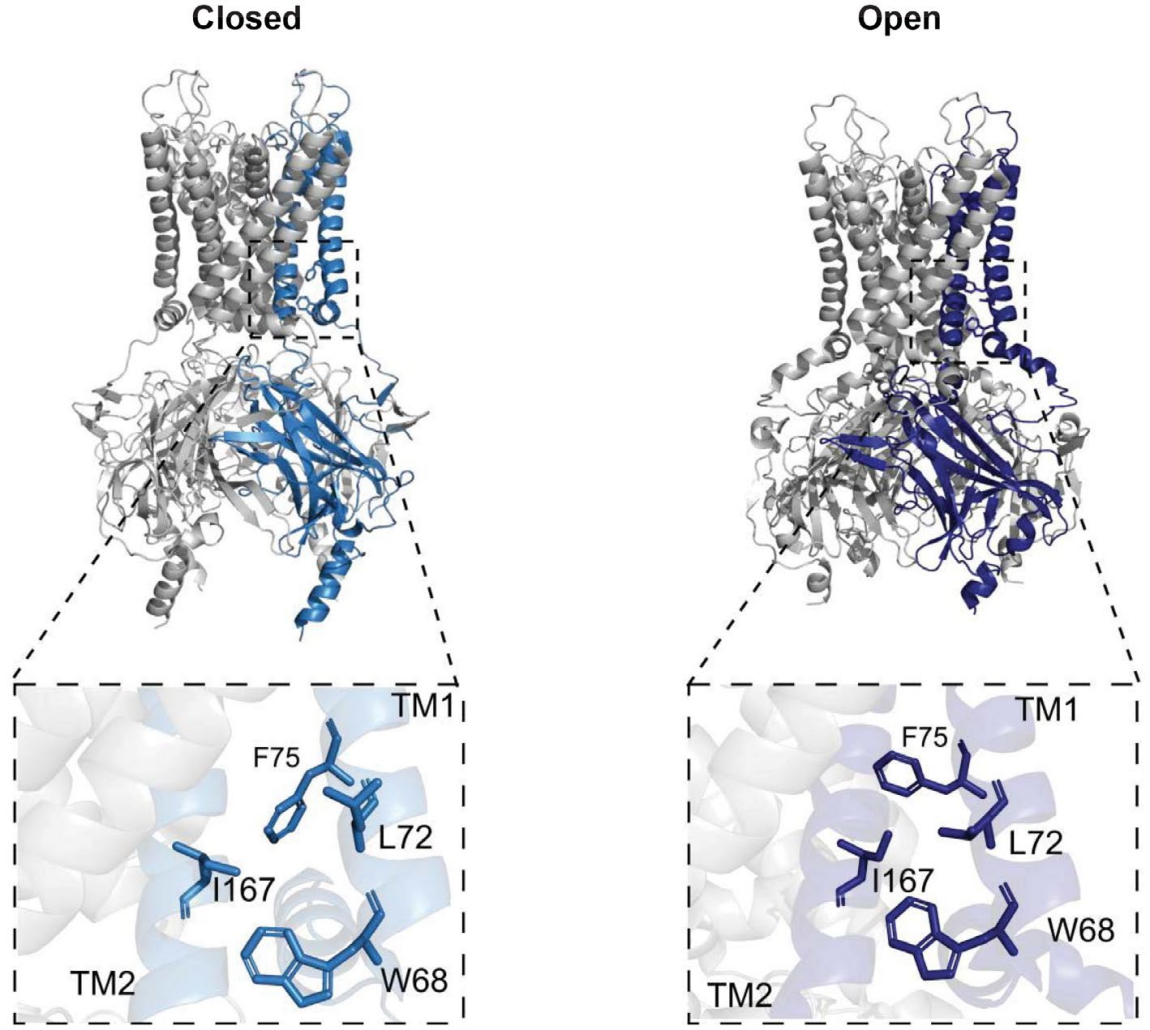
Structural changes between the closed (left, PDB: 6C3O) and open (right, PDB: 7S5T) conformations of Kir6.2. A comparison of the position of TM1 residues in close proximity to I167 in the two conformations is shown.

Next, in order to further investigate the mechanism underpinning the increased *P*_o_ for the I167L mutation, we investigated whether any amino acid changes in the nearby TM1 could potentially ameliorate the increase of *P*_o_ caused by the I167L mutation in TM2. As I167 is projecting away from the central channel pore (Figure 1), it is possible that I167 may be interacting with TM1, which is in close range. A high‐resolution cryoEM structure of Kir6.2 in both the open (PDB ID 7S5T) and the closed (PDB ID 6C3O) states identifies three residues, W68, L72, and F75 in TM1 to be in close proximity to I167 and could therefore potentially interact (Figure 3). Thus, we constructed three single mutants (W68A, L72A, F75A) and three double mutants (W68A/I167L, L72A/I167L and F75A/I167L) of Kir6.2 channels to investigate the possible contribution of these TM1 residues.

**FIGURE 4:**
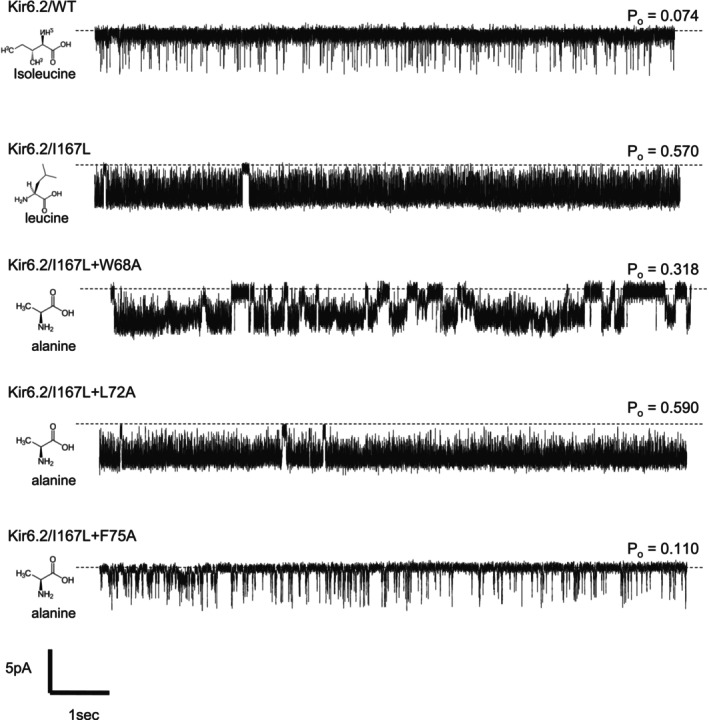
Single channel recordings of Kir6.2 channel of WT, I167L and double mutations with I167L and W68A, L72A and F75A.

The single mutations W68A and F75A showed similar *P*_o_ compared to WT channels (*P*_o_: 0.099 ± 0.045 [*n* = 12] in W68A; *P*_o_: 0.085 ± 0.074 [*n* = 12] in F75A) which would suggest that these residues do not form functionally important contacts with TM2. However, the L72A single mutation showed a significant increase in *P*_o_ compared to WT (*P*_o_: 0.151 ± 0.079 in L72A; *p* < 0.05 compared to WT) (Table 2). The double mutant I167L/ F75A showed a significantly decreased *P*_o_ compared to the I167L single mutation (*P*_o_: 0.11 ± 0.12 [*n* = 6] in I167L/F75A vs. *P*_o_: 0.570 ± 0.239 [*n* = 13] in I167L: *p* < 0.05) (Figure 4). The *P*_o_ values of I167L/W68A showed a moderate but significant decrease compared to the I167L single mutation (*P*_o_: 0.318 ± 0.199 [*n* = 11] in I167L/W68A vs. *P*_o_: 0.570 ± 0.239 [n = 13] in I167L: p < 0.05). However, the *P*_o_ values of I167L/L72A showed a similar *P*_o_ compared to the I167L single mutation (*P*_o_: 0.59 ± 0.246 (n = 12) in I167L/L72A vs. *P*_o_: 0.570 ± 0.239 (n = 13) in I167L: *p* < 0.05) but showed a significant increase in *P*_o_ compared to the L72A single mutation (*P*_o_: 0.59 ± 0.246 (*n* = 12) in I167L/L72A vs. *P*_o_: 0.151 ± 0.079 (*n* = 10) in L72A; *p* < 0.0001) (Table 2).

## 4 DISCUSSION

Residues responsible for gain‐of‐function mutations in Kir6.2 that cause neonatal diabetes are mainly located in two regions: the ATP binding site and the gating region of the channel pore (Shimomura & Maejima, 2017). We have previously demonstrated, using molecular dynamics simulations, that the R50P mutation in the ATP binding site of Kir6.2 destabilizes ATP binding, thereby favoring the stabilization of the channel in the open configuration (Horita et al., 2021).

In Kir6.2 channels, there are three gates that regulate the crossing of K^+^: the cytoplasmic G‐loop, the selectivity filter, and the bundle‐crossing gate (Xie et al., 2007; Jogini et al., 2023). Kir channels are different from other K^+^ channels due to the existence of a large cytoplasmic domain (CTD). This allows ligands such as ATP, G‐proteins, and PIP_2_ to bind to the intracellular domain, inducing conformational changes in the overall channel that regulate channel activity. In Kir channels, the rotational movement of TM2 is considered to be strongly connected with conformational changes in the CTD, which includes the regulation of the opening and closing of the bundle‐crossing gate (Hibino et al., 2010; Bavro et al., 2012). The bundle‐crossing gate of Kir6.2 is also regulated by SURs, and both drugs and nucleotides binding to Kir6.2 and SUR1 subunits can alter channel activity.

However, the exact mechanism for the opening and closing of the bundle‐crossing gate remains unclear. From the structure of Kir6.2, TM2 comprising the bundle‐crossing gate is adjacent to TM1, and it is therefore likely that the interaction of TM1 and TM2 may contribute to the regulation of the opening and closing of the bundle‐crossing gate. As I167 on TM2 is projecting away from the central channel pore (Figure 1), it is possible that I167 may be interacting with TM1, which is in close range. Interestingly, here we show that only the mutations I167L and I167F, but not I167A, I167V, or I167Q, resulted in an increase in *P*_o_. A higher intrinsic open probability is indicative of an increased open state stability, which would suggest that the I167L and I167F mutations stabilize the open state. The mutation of residue 167 from a hydrophobic to hydrophilic amino acid (I167Q) did not alter the *P*_o_, suggesting that the hydrophobicity of residue 167 has a minimal effect on channel activity. Conversely, mutations of residue 167 within hydrophobic amino acids showed either no change (I167V, I167A) or an increase (I167L, I167F) in *P*_o_ compared to WT channel. The precise mechanisms underlying these differences in *P*_o_ remain unclear. However, one possible explanation is the size of the amino acid side chains. The hydrophobic mutations that resulted in increased *P*_o_ have relatively larger side chains compared to those mutations that showed no change in *P*_o_. Further studies are required to elucidate the detailed mechanisms.

Scrutiny of reported open and closed structures of the K_ATP_ channel, suggested three residues in TM1 (W68, L72, and F75) as possible interaction partners of I167 in TM2 (Figure 3). Based on previous structures of other related Kir channels (Nishida et al., 2007; Tao et al., 2009), movement of the nearby residue W68 of Kir6.2 was proposed to play an important role as a gate keeper of the bundle‐crossing gate. It was suggested that the rotational position of W68 could affect channel *P*_o_. (Männikkö et al., 2011). Several previous studies have also implicated W68 and its interaction with K170 in both ATP sensitivity and *P*_o_ of Kir6.2 (Zhang et al., 2015). Our data demonstrated that single mutation in W68 (W68A) and F75 (F75A) did not affect the *P*_o_, suggesting mutation of I167 to be crucial to the disruption of channel activity. However, the single mutation of L72A resulted in a slight but significant increase in *P*_o_ compared to the WT. This increase in *P*_o_ was further enhanced by introducing an additional mutation of I167L (I167L + L72A), reaching the same level as the single mutation of I167L. This indicates that while the L72A mutation alone may contribute to increased channel *P*_o_, the single mutation of I167L can override the effect of L72A and induce a further increase in *P*_o_.

Our data suggests that there is indeed a functional interaction of these amino acids between TM1 and TM2, and specifically, the double I167L‐F75A mutation had a dramatic reversal of increased open probability back to WT levels. A closer look at the structural arrangement of residues I167, W68, and F75, comparing recent high‐resolution structures of Kir6.2, shows that W68 and F75 are forming a hydrophobic environment around I167 (Figure 3), suggesting an important role of this hydrophobic cluster in controlling channel gating. Further, the residue F75 appears to adopt different rotamers in the closed and open conformations of Kir6.2. Therefore, it could be that the mutations I167L and I167F stabilize the rotamer of F75 adopted in the open conformation, similar to that which is seen in the isoleucine to leucine mutation of KlenTaq1 DNA polymerase, which causes major structural rearrangements and results in cold sensitivity of the protein (Wu et al., 2014). Taken together, the data suggests that the mutations shown here destabilize the closed channel state by disrupting the closed‐state hydrophobic interactions of the helix cross bundle.

It is important to note that a limitation of the study is that this work was done in the absence of SUR1, and any conclusion drawn from this work should be repeated with the intact octameric complex. However, the main aim of this work was to investigate the interactions between TM1 and TM2 as well as DEND mutations on Kir6.2, which are unaffected by sulfonylurea drugs, in which channel gating appears to be uncoupled from SUR.

## 5 CONCLUSIONS

It is well established that sulfonylurea sensitivity is decreased in K_ATP_ channel mutations which increase *P*_o_. The data presented here show that interactions between TM1 and TM2 may contribute to the regulation of the opening and closing of the bundle‐crossing gate of K_ATP_ channels and may be an important target for drug development. A detailed understanding of the molecular forces which regulate K_ATP_ channel opening and closing is essential for the development of drugs to treat DEND patients refractory to sulfonylureas.

## AUTHOR CONTRIBUTIONS

Ryoko Kawashima performed the experiments and wrote the draft. Shoichiro Horita contributed only to the preparation of viral vector stocks and performed cell transfections. Shizu Hidema and Shingen Misaka, Shigeki Kato, Manabu Suzuki, and Kazuto Kobayashi designed the viral vector, performed electrophysiological studies, and analyzed the data. Mitsusaki Hosoya and Hayato Go supervised the work and edited the manuscript. Heidi de Wet designed the experiments, interpreted the data, and revised the paper. Yuko Maejima and Charlotte Meller analyzed and interpreted the data, drafted, and revised the paper. Kenju Shimomura performed electrophysiology experiments, analyzed, interpreted data, designed, and supervised the study, and revised the paper. All authors agree to be accountable for all aspects of the work. All authors have read and approved the final version of the manuscript.

## CONFLICT OF INTEREST STATEMENT

The authors declare no conflict of interest.

## ETHICS STATEMENT

Our work only used culture cell lines. Therefore, this article does not contain any data relevant to ethics approval statement.

## FUNDING INFORMATION

This work was supported by Grant‐Aid for Scientific Research © (18K08483 to YM, 26461366 to KS).

## Data Availability

The data that support the findings of this study are available from the corresponding author upon reasonable request.
